# The ethics of simplification: balancing patient autonomy, comprehension, and accuracy in AI-generated radiology reports

**DOI:** 10.1186/s12910-025-01285-3

**Published:** 2025-10-15

**Authors:** Hong-Seon Lee, Seung-Hyun Song, Chaeri Park, Jeongrok Seo, Won Hwa Kim, Jaeil Kim, Sungjun Kim, Kyunghwa Han, Young Han Lee

**Affiliations:** 1 Department of Radiology, Gangnam Severance Hospital, Yonsei University College of Medicine, Seoul, Republic of Korea; 2 BeamWorks Inc, Daegu, Republic of Korea; 3 Department of Radiology, School of Medicine, Kyungpook National University, Kyungpook National University Chilgok Hospital, Daegu, Republic of Korea; 4 School of Computer Science and Engineering, Kyungpook National University, Daegu, Republic of Korea; 5 Department of Medical Device Engineering and Management, The Graduate School, Yonsei University College of Medicine, Seoul, Republic of Korea; 6 Department of Integrative Medicine, The Graduate School, Yonsei University College of Medicine, Seoul, Republic of Korea; 7 Institute for Innovation in Digital Healthcare, Yonsei University, Seoul, Republic of Korea; 8 Department of Radiology, Research Institute of Radiological Science and Center for Clinical Imaging Data Science, Severance Hospital, Yonsei University College of Medicine, 50-1, Yonsei-ro, Seodaemun-gu, Seoul, 03722 Republic of Korea

**Keywords:** AI-generated radiology reports, Large Language Models, Readability and comprehension, Clinical accuracy, Patient autonomy, Informed consent, Ethical implications

## Abstract

**Background:**

Large language models (LLMs) such as GPT-4 are increasingly used to simplify radiology reports and improve patient comprehension. However, excessive simplification may undermine informed consent and autonomy by compromising clinical accuracy. This study investigates the ethical implications of readability thresholds in AI-generated radiology reports, identifying the minimum reading level at which clinical accuracy is preserved.

**Methods:**

We retrospectively analyzed 500 computed tomography and magnetic resonance imaging reports from a tertiary hospital. Each report was transformed into 17 versions (reading grade levels 1–17) using GPT-4 Turbo. Readability metrics and word counts were calculated for each version. Clinical accuracy was evaluated using radiologist assessments and PubMed-BERTScore. We identified the first grade level at which a statistically significant decline in accuracy occurred, determining the lowest level that preserved both accuracy and readability. We further assessed potential clinical consequences in reports simplified to the 7th-grade level.

**Results:**

Readability scores showed strong correlation with prompted reading levels (*r* = 0.80–0.84). Accuracy remained stable across grades 13–11 but declined significantly below grade 11. At the 7th-grade level, 20% of reports contained inaccuracies with potential to alter patient management, primarily due to omission, incorrect conversion, or inappropriate generalization. The 11th-grade level emerged as the current lower bound for preserving accuracy in LLM-generated radiology reports.

**Conclusions:**

Our findings highlight an ethical tension between improving readability and maintaining clinical accuracy. While 7th-grade readability remains an ethical ideal, current AI tools cannot reliably produce accurate reports below the 11th-grade level. Ethical implementation of AI-generated reporting should include layered communication strategies and model transparency to safeguard patient autonomy and comprehension.

## Introduction

The incorporation of cutting-edge large language models (LLMs) such as ChatGPT and Google Gemini into medical records, especially radiology reports, represents a significant advancement toward making medical information more accessible and understandable to patients [1–3]. Historically, radiology reports have utilized highly technical and professional jargon tailored to healthcare providers, creating a communication gap that limits patient understanding, participation, and autonomy in healthcare management [4–9]. However, recent transformations in healthcare delivery, including the rapid expansion of telemedicine, widespread adoption of patient portals, and pivotal regulatory mandates such as the 21st Century Cures Act, have fundamentally altered patients’ interactions with their electronic health records, positioning them as active participants in their care rather than passive recipients of medical information [10–12].

This shift toward increased patient access underscores ethical obligations in healthcare communication, specifically regarding patient autonomy, informed consent, transparency, and equity in information dissemination. Patient autonomy, defined as the patient’s right and capacity to make informed decisions regarding their own health, requires transparent and understandable communication. Informed consent necessitates that patients have sufficient and accurate information to voluntarily consent to or refuse medical interventions based on clear comprehension of benefits, risks, and alternatives. The participatory medicine movement, exemplified by the e-patient concept, actively advocates for patient empowerment, engagement, and collaborative decision-making between patients and healthcare providers (https://participatorymedicine.org/, March 21, 2025). Promoting patient participation through clearer communication has tangible ethical benefits, significantly reducing preventable patient safety incidents by fostering transparency and trust [13, 14]. Accurate, comprehensible information tailored to patients’ health literacy is not merely beneficial but ethically essential, as miscommunication and misunderstanding directly impact the quality of care, patient autonomy, and informed decision-making [15–17].

Nevertheless, critical ethical and legal considerations arise when simplifying complex medical terminology [18]. Regulatory and ethical frameworks require healthcare providers to ensure informed consent through clear and accurate communication. Although readability guidelines traditionally recommend that patient-facing materials be simplified to below a 7th-grade reading level [12, 19], this benchmark is supported by national recommendations: the U.S. National Institutes of Health advises using a 6th–7th grade level to ensure broad comprehension [20], and the American Medical Association manual similarly recommends 5th–6th grade readability for clinical materials [21]. Nevertheless, overly simplified medical reports might unintentionally omit or distort essential information, violating ethical responsibilities of accuracy, transparency, and patient autonomy [22, 23]. Consequently, the ethical imperative to balance readability and accuracy in patient-centered reporting necessitates rigorous examination and clear ethical standards for deploying LLM-based simplifications.

The advent of advanced LLMs introduces new possibilities for addressing this challenge. With high linguistic coherence and contextual understanding, these artificial intelligence (AI) models can translate medical jargon into accessible narratives without severely distorting original meanings [24]. Efforts to simplify radiology reports using LLMs aim to improve patients’ access to medical information and promote patient-centered decision-making [25]. Yet, ethical caution is required because improper implementation may inadvertently diminish patient autonomy or undermine informed consent through the loss of critical clinical detail. In this study, we hypothesize that there exists a pragmatic lower bound to readability, an inflection point where further simplification significantly compromises the accuracy of radiology reports. Below this threshold, we argue, ethical harms emerge: misinformation, degraded informed consent, impaired autonomy, and increased liability. Thus, we seek to identify the current accuracy-preserving threshold using expert assessments and semantic similarity metrics across 17 grade levels of LLM-generated radiology reports.

Our aim is not to redefine what the ideal readability level should be, but to confront the ethical consequences of what current technology can and cannot achieve. We therefore pose the following question: “At what readability level do AI-generated radiology reports begin to compromise clinical accuracy to a degree that undermines ethical standards for patient communication?” Far from being a purely technical issue, this question speaks to foundational bioethical concerns: the patient–clinician relationship, information asymmetry, and the integrity of consent in the age of artificial intelligence.

This study further addresses epistemic injustice [26–29], where simplified AI-generated reports risk marginalizing patients’ access to nuanced clinical information, potentially limiting their understanding and decision-making capabilities. Additionally, considering relational autonomy [30–32], where patients’ choices are influenced by their social interactions and contexts, providing sufficient and contextually sensitive information becomes ethically critical.

## Materials and methods

### Data selection

**Fig. 1:**
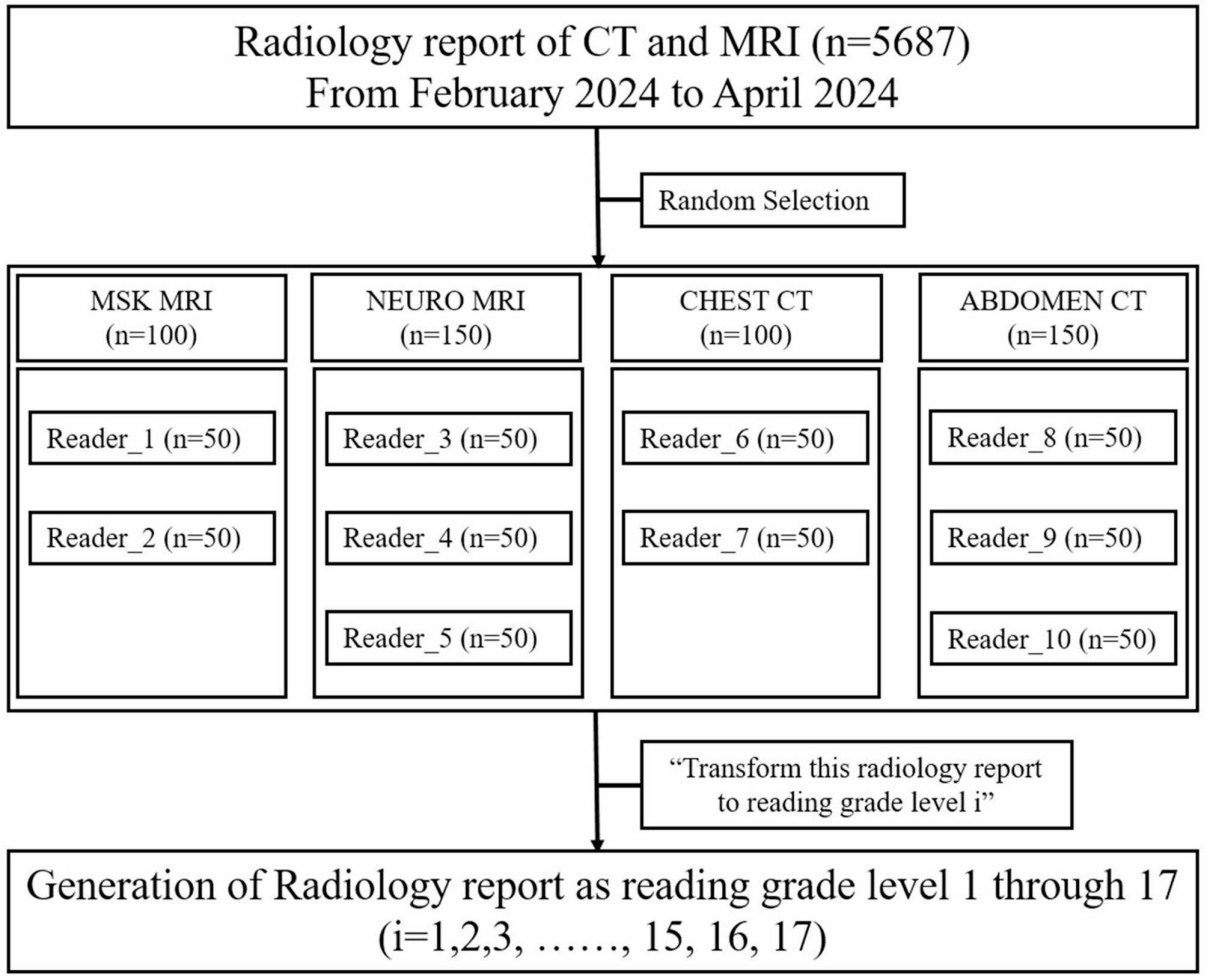
Flow chart of data selection

This retrospective study was conducted at a single tertiary hospital after receiving approval from the institutional review board of our hospital. A waiver of informed consent was granted owing to the retrospective nature of the study. This study complied with the Declaration of Helsinki and the Health Insurance Portability and Accountability Act. A data selection flowchart is provided in Fig. 1.

From February 18 to April 9, 2024, all computed tomography (CT) and magnetic resonance imaging (MRI) scans performed and interpreted, totalling 5,687 cases, were eligible. From these 5,687 scans, we randomly sampled 500 cases using the random number generator in Excel 365 (Microsoft Corporation, Redmond, WA), assigning 50 cases to each of 10 radiologists. As a result, 500 radiology reports from 10 radiologists, each with a minimum of 5 years of experience in their respective specialties, were analysed. The specialists included two musculoskeletal radiologists, three neuroradiologists, two chest radiologists, and three abdominal radiologists. Consequently, 500 scans were selected regardless of inpatient or outpatient status: 100 chest CTs, 150 abdominal CTs, 100 musculoskeletal (including spine and joint) MRIs, and 150 neurological (including brain and neck) MRIs. Ultrasound, mammography, and X-rays were excluded owing to the specific characteristics associated with each modality. Ultrasound examinations were omitted because they involved direct patient interactions, allowing for an immediate explanation of the findings to the patients. The study hospital generally does not utilize a standardized reporting system; thus, most reports are composed in free text. However, mammography reports were excluded because of their typically well-structured format, which renders them less suitable for simplification. Furthermore, radiographs were considered inappropriate for inclusion because national clinical practice patterns necessitate reading a large volume of scans in a short period, typically resulting in brief reports consisting of three sentences or fewer. These conditions were deemed unsuitable for simplifying reporting in our study.

### Report generation using an LLM (ChatGPT-4)

Patient identification codes were removed to ensure compliance with Health Insurance Portability and Accountability Act regulations. For preparation, headers, which encompassed clinical information, MRI sequence details, and comparisons with previous studies, were removed from all radiology reports. No further modifications were made to these reports. The input radiological reports contained findings, impressions, and recommendations.

The OpenAI API, specifically the GPT-4 Turbo model (version: GPT-4-turbo-2024-04-09) developed by OpenAI (San Francisco, CA), was employed. The prompt “Transform this radiology report to reading grade level *i*” was crafted to instruct the model. Each radiology report was transformed into 17 versions (*i* = 1, 2, 3, …, 15, 16, and 17) corresponding to reading grades 1–17. The original report’s maximum average readability score was 19.94, with 10 cases (2.0%) scoring 18 or higher. However, 6.8% of cases scored 17 or higher, indicating that setting the maximum reading grade level at 17 would cover all significant levels.

We did not engage in formal prompt engineering. However, careful consideration was given to the wording of the prompt to ensure that the transformation process maintained the accuracy and integrity of the original medical information. Specifically, instead of using the word “simplify,” we opted for “transform” in the prompt (e.g., “Transform this radiology report to a 7th-grade reading level”). This choice was made to avoid the risk of oversimplifying the content, thereby preventing the loss of critical medical details. The model temperature was set to the default value of 0.7. The temperature setting controls the randomness of a language model’s responses. Lower temperatures produce more predictable and focused answers, while higher temperatures result in more creative and varied outputs. Python (version 3.12.3), developed by the Python Software Foundation (Wilmington, DE), was used for scripting and automation. Jupyter Notebook (version 6.5.4), a product of Project Jupyter (San Diego, CA), was employed for interactive coding and documentation. The analysis was conducted on a Windows 10 workstation (version 10.0.19042; Microsoft Corporation, Redmond, WA) utilizing Anaconda (version 2024.02-1, Austin, TX) for package management and environmental setup.

### Validation of transformed radiology reports

To verify that the transformed radiology reports matched each reading grade level, we calculated the Flesch–Kincaid readability score (Flesch 2007) [33], Gunning fog score [34], simple measure of Gobbledygook score [35], automated readability index score [36], and word count. These scores correspond to the reading grade levels of American students in their respective [33–36]. We then analyzed the correlation between these metrics of the transformed reports and the reading grade levels requested from the LLM, using Spearman’s correlation coefficient to evaluate how closely the prompted and generated reading levels matched, compared to an ideal scenario where y = x (i.e., the prompted and generated levels perfectly align).

### Accuracy test for transformed radiology reports

To assess the quality of the transformed radiology reports, an evaluation process was conducted by the radiologists who created the original reports and PubMed-BERTScore [37]. Each of the 10 radiologists evaluated a randomly selected set of 10 reports from their set of 50. Each evaluation was conducted independently. The paraphrased texts were evaluated based on the following criteria:

- Meaning accuracy (score range: 1–5): Does the paraphrased text convey the same meaning as the original?
- Word choice (score range: 1–5): Are the words used appropriately and accurately?
- Grammar and sentence structure (score range: 1–5): Is the paraphrased text grammatically correct and well-structured?

The total score for each transformed text was calculated by summing the individual scores for meaning accuracy, word choice, and grammar and sentence structure. The intra-rater reliability of meaning accuracy, word choice, grammar and sentence structure, and total score were assessed with a 2-month time interval between evaluations.

We also assessed whether the transformation of original reports to the 7th-grade reading level, the target for patient-centered reports, could lead to potential changes in patient management. We considered a potential change to be present if the transformation affected the need for appropriate follow-up, altered the necessity or method of surgery or treatment, or led to different management decisions. Additionally, we analyzed the inaccuracy patterns in cases that could potentially lead to changes in patient management. The analysis was categorized into three overlapping patterns: inappropriate generalization, omission, and incorrect conversion. Inappropriate generalization refers to the use of a hypernym that inappropriately distorts the original meaning of a specific disease or anatomical term. Omission occurs when crucial information is left out. Incorrect conversion refers to cases where important information is altered in a way that changes the meaning entirely, rather than just generalizing it.

BERTScore is a metric used for evaluating the quality of text generated by natural language processing models. It leverages Bidirectional Encoder Representations from Transformers, a powerful language representation model, to assess the similarity between a generated text and a reference text [38]. We used the PubMed-BERTScore, specifically trained on 14 million PubMed abstracts, to evaluate the accuracy of paraphrased sentences compared to the original sentences. Unlike the traditional BERTScore [38], PubMed-BERTScore is specifically trained from the ground up using medical domain vocabulary, allowing it to more accurately understand and process medical terms. This model includes a vocabulary that is finely tuned to recognize important biomedical terms, making it much more effective at handling information related to diseases, drugs, and genes.

### Exploration of appropriate reading grade level

To determine the appropriate reading grade level that balances accuracy and readability, we first established the threshold for an accurate report as the median of total accuracy scores based on radiologists’ assessments and the PubMed BERTScore across reading grade levels 1–13. We opted for levels 1–13 instead of 1–17. Grade 13 was chosen as the baseline for two primary reasons. First, the average readability score of our original radiology reports was 13.3, so selecting a higher baseline would not align with our objective of observing changes in accuracy during the simplification process. Second, setting the baseline at grade 13 allowed us to capture the maximum level of detail and specificity before any simplification occurred, ensuring that the baseline content was as rich and informative as possible, crucial for evaluating the impact of text simplification. Consequently, we compared the paraphrased radiology reports starting from reading grade level 13 and descending through levels 12, 11, 10, and so on, with level 13 as the baseline point. We identified the first level that exhibited a statistically significant difference, and then selected the level just above it as the appropriate level that preserves accuracy while enhancing readability.

### Statistical analysis

A non-parametric measure of correlation was used because the data did not meet the assumptions of normality required for parametric tests. Spearman’s correlation coefficients were calculated to assess the strength and direction of the relationships between the reading grade levels and each readability score and accuracy metric. The interpretation of the magnitude of the Spearman correlation coefficient can be generally categorized as follows: 0.00 to 0.19: very weak or no correlation; 0.20 to 0.39: weak correlation; 0.40 to 0.59: moderate correlation; 0.60 to 0.79: strong correlation; 0.80 to 1.00: very strong correlation. To measure intra-rater reliability for meaning accuracy, word choice, grammar and sentence structure, and total score, intraclass correlation coefficients (ICCs) were used. The ICCs were interpreted as follows: values below 0 indicated no reliability, 0.01–0.20 indicated slight reliability, 0.21–0.40 indicated fair reliability, 0.41–0.60 indicated moderate reliability, 0.61–0.80 indicated substantial reliability, and 0.81–1.00 indicated almost perfect reliability. To assess the differences between the total accuracy scores and PubMedBERTScore across the reading grade levels, we employed the Wilcoxon signed-rank test. The *p*-values were adjusted by multiplying by 136, considering a combination of _17_C_2_. We conducted McNemar’s test to assess the statistical significance of differences between paired proportions of accurate reports. This test was chosen owing to its suitability for analyzing dichotomous outcomes from related samples, such as comparing the accuracy of different reading grade levels. The threshold for statistical significance was set at *p* < 0.05. Python version 3.12.3 was used to collect readability scores and word counts, as well as for data visualization and statistical analyses.

## Results

### Readability of original reports

Only 5% (5/100) of the reports evaluated for accuracy were negative reports. These included three brain MRIs for metastasis screening and two cardiac CTs performed on young patients. Among the 500 radiology reports analyzed, 71.2% (356/500) originated from outpatient settings, 18.8% (94/500) from inpatient settings, 9% (45/500) from the emergency room, and 1% (5/500) from the intensive care unit. The readability scores and word counts are summarized in Table 1. The median (Q1–Q3) Flesch–Kincaid grade was recorded at 11.7 (10.0–13.2), Gunning fog index at 13.8 (12.4–15.3), simple measure of Gobbledygook index at 13.7 (12.7–14.8), and automated readability index at 14.2 (11.9–16.6). The overall median readability score was 13.3 (11.8–14.8). The median word count was 88.0 (81.0–120). Depending on the reader, the median readability score varied from 11.4 to 16.1, and the median word count ranged from 59.0 to 118.5. By category, the median readability score ranged from 12.0 to 14.2, and the median word count ranged from 79.5 to 94.0.

**Table 1:** Readability of original reports

	Flesch Kincaid grade	Gunning Fog index	SMOG* index	Automated readability index	Average	Words count
Overall	11.68 (2.34)	13.94 (2.38)	13.64 (2.01)	14.56 (3.37)	13.39 (2.77)	82.26 (28.13)
Reader_1	12.55 (2.66)	13.45 (2.12)	14.03 (1.80)	16.65 (2.88)	14.17 (2.83)	88.62 (39.47)
Reader_2	12.33 (2.50)	13.76 (2.70)	13.23 (2.79)	16.75 (2.73)	14.02 (3.14)	69.14 (24.50)
Reader_3	10.78 (2.05)	14.26 (2.63)	12.02 (3.53)	11.30 (2.44)	12.09 (3.00)	49.22 (7.90)
Reader_4	11.96 (2.05)	13.82 (2.32)	14.78 (1.48)	14.82 (2.90)	13.84 (2.52)	104.56 (22.27)
Reader_5	14.10 (1.59)	16.41 (2.03)	15.43 (0.91)	18.36 (2.23)	16.07 (2.34)	87.26 (21.00)
Reader_6	11.39 (1.88)	14.10 (2.37)	13.48 (1.02)	14.23 (2.43)	13.30 (2.29)	81.28 (29.27)
Reader_7	12.47 (2.31)	14.44 (1.88)	14.04 (1.23)	13.58 (2.97)	13.63 (2.30)	77.62 (14.36)
Reader_8	10.91 (1.67)	13.85 (1.74)	13.46 (1.01)	13.02 (1.85)	12.81 (1.96)	110.14 (19.43)
Reader_9	10.31 (1.72)	13.16 (1.70)	13.12 (1.07)	12.72 (2.28)	12.33 (2.10)	88.82 (18.36)
Reader_10	10.01 (1.71)	12.15 (1.92)	12.73 (1.05)	11.51 (2.51)	11.6 (2.11)	65.98 (11.36)
MSK	12.44 (2.57)	13.60 (2.42)	13.64 (2.37)	16.70 (2.79)	14.10 (2.99)	78.88 (34.12)
NEURO	12.28 (2.34)	14.83 (2.58)	14.08 (2.70)	14.83 (3.84)	14.00(3.10)	80.34 (29.44)
ABDOMEN	10.41 (1.73)	13.06 (1.91)	13.11 (1.08)	12.42 (2.31)	12.25 (2.11)	88.31 (24.59)
CHEST	11.93 (2.16)	14.27 (2.14)	13.76 (1.16)	13.91 (2.72)	13.47 (2.30)	79.45 (23.01)

*SMOG Simple Measure of Gobbledygook

### Correlation of generated radiology reports to readability score

**Fig. 2:**
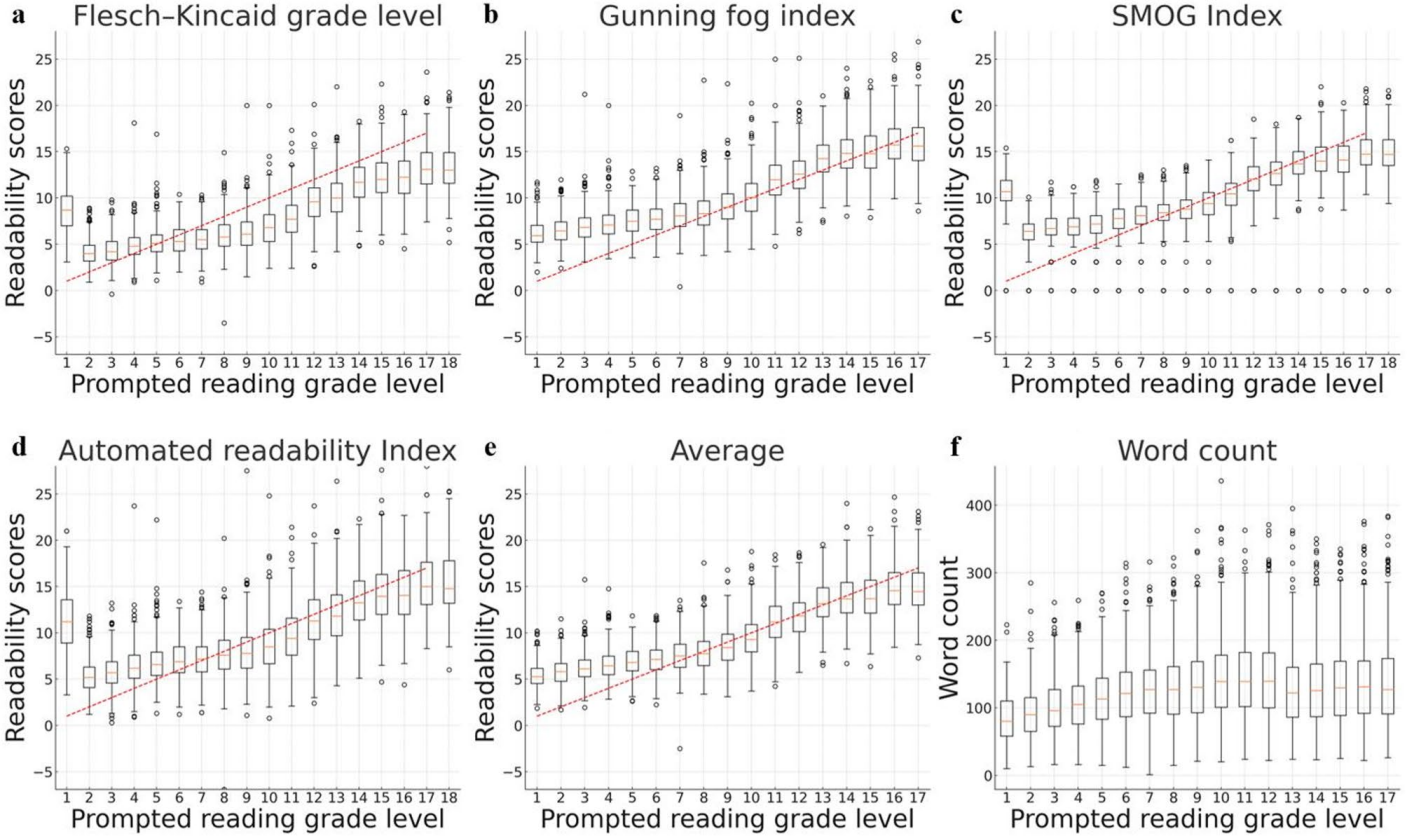
Correlation of generated radiology reports to readability score. Each graph represents median and interquartile range of Flesch–Kincaid grade (**a**), Gunning fog (**b**), SMOG index (**c**), automated readability index (**d**), average readability score (**e**) and word count (**f**) according to prompted reading grade level (from 1 to 17). Boxplots illustrate how these readability metrics and word count vary across different grade levels. For readability scores, correlation coefficients were found to be very strong, ranging from 0.9751 to 0.9975. This indicates high degree of linear relationship between readability scores and reading grade levels. However, correlation coefficient for word count was relatively lower at 0.7069. For word count, there was a slight decrease between reading grade levels 12 and 13, followed by a plateau beyond grade level 13. This trend indicates that word count stabilizes and shows minimal variation at higher reading grade levels

Figure 2 depicts the correlation between the readability metrics and prompted reading grade levels from 1 to 17 for the generated radiology reports. As the prompted reading grade level increased, all readability scores similarly trended upward, signifying that the complexity of the text intensified. Furthermore, the word counts typically increased with the prompted reading grade level, albeit with greater variability than that observed in the readability scores. The readability scores exhibited a high correlation with the reading grade level, ranging from 0.80 to 0.84, indicating a very strong relationship. In contrast, while the word count also increased as the reading grade level increased, it demonstrated more variability and a weak correlation of 0.25 with the prompted reading grade level, suggesting less consistency compared to the readability scores.

### Total score of accuracy by radiologists and PubMed-BERTScore

**Fig. 3:**
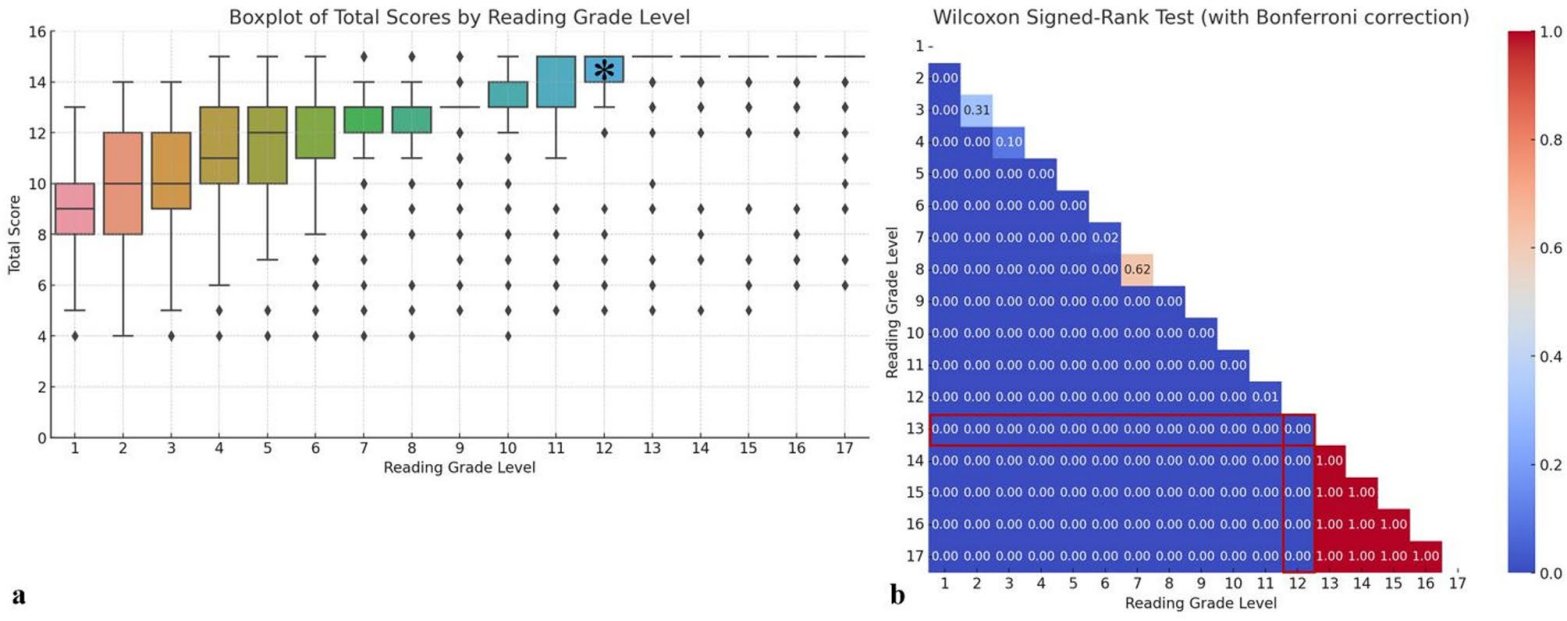
Total score of accuracy by Radiologists. Graph **a** represents median and interquartile range of total accuracy score evaluated by radiologists according to reading grade level. Graph **b** is a heatmap displaying *p*-values obtained from Wilcoxon signed-rank tests for all pairwise combinations of reading grade levels from 1 to 17 (_17_C_2_ = 136). Using the average readability score of the original report, which is closest to the 13th reading grade level, as a reference, it was found that the accuracy significantly decreased at the level immediately below, level 12, indicated by asterisks and highlighted in red

The graph demonstrates that higher reading grade levels were associated with incrementally higher total scores up to a certain threshold (Fig. 3). At reading grade level 13, there were no significant differences in accuracy between levels 17, 16, 15, and 14 (all *p* > 0.9999). However, there was a significant difference between levels 13 and 12, with median (Q1–Q3) values of 5.0 (5.0–5.0) and 5.0 (3.0–5.0), respectively (*p* < 0.0001). A plateau in accuracy was achieved between levels 13 and 17 with a median total score of 15. The ICC for the total score was 0.81. Other detailed accuracy measures, including meaning accuracy, word choice, and grammar and sentence structure, were 0.88, 0.77, and 0.71, respectively, indicating substantial to almost perfect reliability (all *p* < 0.001).

**Fig. 4:**
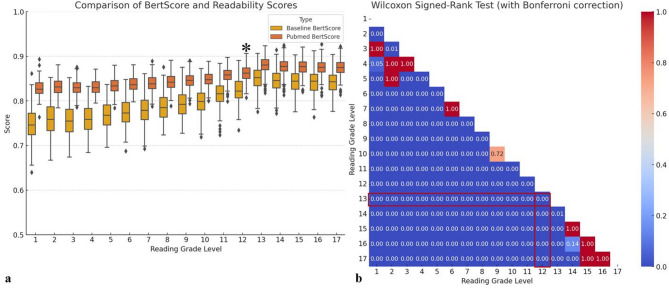
Total score of accuracy by PubMed-BERTScore. These graphs represent median and interquartile range of total score evaluated by both traditional (baseline) BERTScore and PubMed-BERTScore according to reading grade level (**a**). Graph **b** is a heatmap displaying *p*-values obtained from Wilcoxon signed-rank tests for all pairwise combinations of reading grade levels from 1 to 17 (_17_C_2_ = 136) in PubMed-BERTScore. Using level 13 as a reference, it was observed that the accuracy significantly decreased at the immediately lower level, level 12, as indicated by asterisk and highlighted in red

In analyses using traditional BERTScore and PubMed-BERTScore (Fig. 4), level 13 recorded the highest scores with median (Q1–Q3) values of 0.8519 (0.8342–0.8687) and 0.8803 (0.8697–0.8927), respectively. Scores for both metrics decreased gradually as the levels diverged from 13. Notably, scores from PubMed-BERTScore were significantly higher than those from traditional BERTScore (median [Q1–Q3], 0.8505 [0.8320–0.8705] vs. 0.8003 [0.7651–0.8341], *p* < 0.001). A descending-order comparison from level 13 in the PubMed-BERTScore graph showed significant differences at all levels (all *p* < 0.0001), including level 12 (asterisk), where the median (Q1–Q3) values were 0.8803 (0.8697–0.8927) and 0.8623 (0.8502–0.8739), respectively (*p* < 0.001).

### Exploring the appropriate reading grade level balancing readability and accuracy of radiology reports

**Fig. 5:**
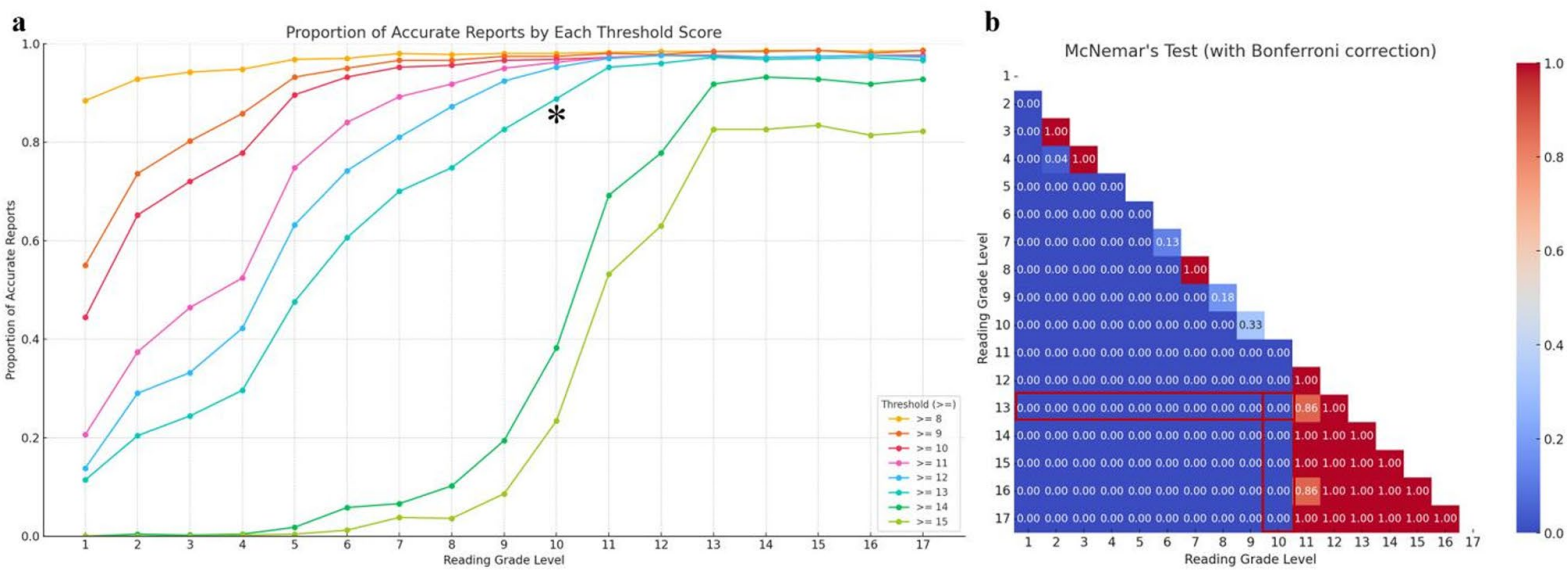
Optimization balancing readability and accuracy using total score assigned by radiologist. The graph **a** delineates the proportion of accurate reports that either meet or exceed various scoring thresholds, denoted as ≥ 8, ≥ 9, ≥ 10, ≥ 11, ≥ 12, ≥ 13, ≥ 14, and ≥ 15. For instance, the line labeled ‘≥ 13’ quantifies the percentage of accurate reports that achieve a score of 13 or higher. Graph **b** is a heatmap that displays *p*-values obtained from Wilcoxon signed-rank tests for all pairwise combinations of reading grade levels from 1 to 17 (a total of 136 combinations) at a threshold level of ≥ 13. The value 13 represents the median total score of paraphrased reports from levels 1 to 13. When comparing statistical significance in descending order from the reference level of 13, the first level to show a significant difference was level 10. Therefore, the optimal level that maintained accuracy while having the lowest readability was level 11 (asterisk and highlighted in red)

**Fig. 6:**
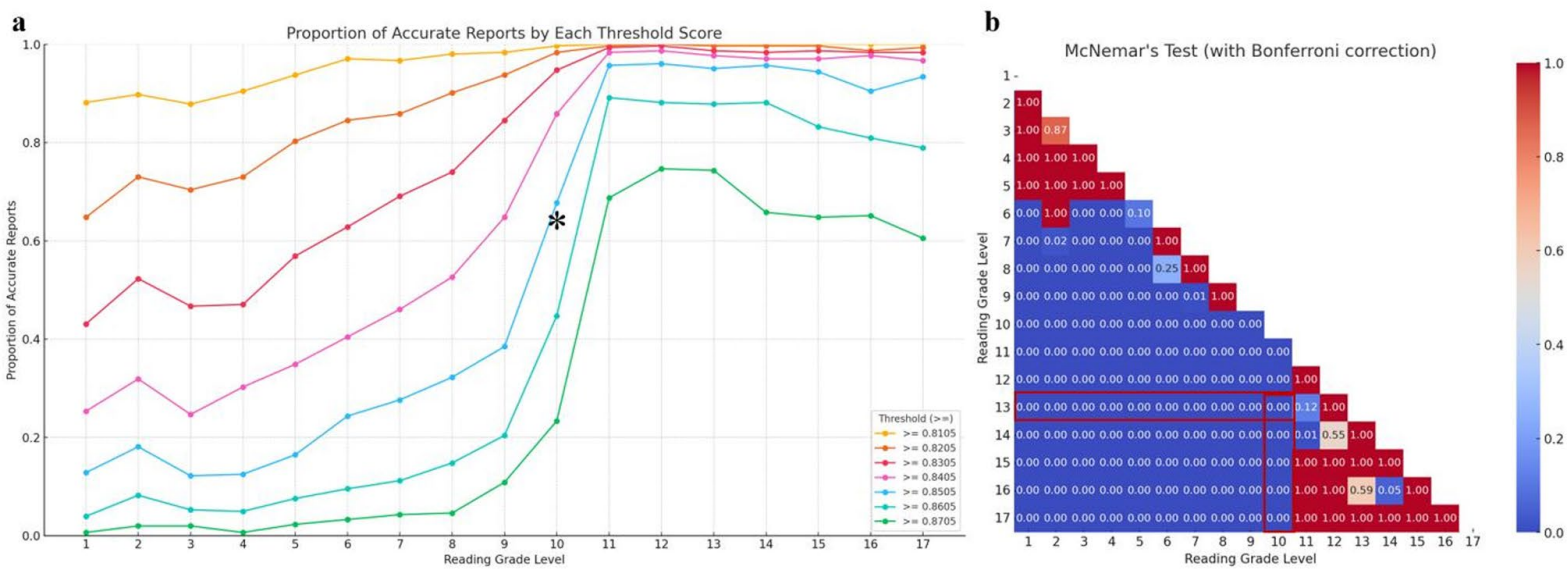
Optimization balancing readability and accuracy using PubMed-BERTScore. The graph **a** delineates the proportion of accurate reports that either meet or exceed various scoring thresholds, denoted as ≥ 0.8105, ≥ 0.8205, ≥ 0.8305, ≥ 0.8405, ≥ 0.8505, ≥ 0.8605, and ≥ 0.8705. For instance, the line labeled ‘≥ 0.8505’ quantifies the percentage of accurate reports that achieve a score of 0.8505 or higher. Graph **b** is a heatmap that displays *p*-values obtained from Wilcoxon signed-rank tests for all pairwise combinations of reading grade levels from 1 to 17 (a total of 136 combinations) at a threshold level of ≥ 0.8505. The value 0.8505 represents the median PubMed-BERTScore of paraphrased reports from levels 1 to 13. When comparing statistical significance in descending order from the reference level of 13, the first level to show a significant difference was level 10. Therefore, the optimal level that maintained accuracy while having the lowest readability was level 11 (asterisk and highlighted in red)

When investigating the statistical significance of the proportion of accurate reports in descending order from reading grade level 13, the first level to show a significant difference was level 10 for both the total accuracy score assigned by radiologists (Fig. 5) and the PubMed-BERTScore (Fig. 6), with results of 0.97 vs. 0.89 and 0.95 vs. 0.68, respectively (all *p =* 0.0001), indicated by an asterisk and highlighted in red. Examples of the original and transformed reports are described in Table 2.

**Table 2:** Examples of original reports and transformed reports

	Original report	Transformed report as 7th reading grade level	Transformed report as 11th reading grade level	Transformed report as 13th reading grade level
Text	1. C1 posterior neural arch fracture, Rt. - Posterior subluxation of the C1/C2, Lt. atlantoaxial joint 2. Recent compression fracture, T3 vertebral body 3. Spinal cord compression, C3-4 and C4-5 - By bulging disc 4. Degenerative spondylolisthesis, C3/4.5 5. Incidentally found crowned dens syndrome - Hypertrophy of soft tissue around odontoid process - Mineralization around the odontoid process (probable CPPD deposition) 6. Degenerative spondylolisthesis, L4/5 - Associated central canal stenosis and bilateral neural foraminal stenosis 7. Bulging disc and ligamentum flavum hypertrophy, L2-3-4 levels 8. Old compression of T8, T10, T12 vertebral bodies	1. There’s a crack in the first bone of the neck and things aren’t lining up right there 2. There’s a newer squished bone in the upper back’s third bone 3. The nerves* in the neck are being pressed because of discs that are sticking out at the neck’s third and fourth bones 4. The neck bone at the third level is slipping over the one below it because of aging 5. There’s a finding we didn’t expect about extra bone growth and hardening around a neck bone** 6. Another neck*** bone sliding over another due to aging at the lower back between the fourth and fifth bones, which also causes narrow spaces for nerves 7. Discs bulging out and thickening ligaments in the lower back affecting several bones 8. Older squished bones in the middle back at three different levels	1. There is a break in the posterior arch of the first cervical vertebra on the right side, causing misalignment of the first and second cervical vertebrae at the left atlantoaxial joint 2. There is a new compression fracture in the third thoracic vertebra 3. The spinal cord is being compressed at the third and fourth cervical levels, as well as the fourth and fifth cervical levels, due to discs that are protruding 4. There is a condition known as degenerative spondylolisthesis at the third and fourth cervical level, where one vertebra slips forward over another 5. A condition called crowned dens syndrome was incidentally discovered, characterized by excessive growth of soft tissue around the odontoid process and mineral deposits around the process, which is likely from calcium pyrophosphate deposition disease 6. A similar forward slippage of the vertebra (degenerative spondylolisthesis) is also seen at the fourth and fifth lumbar levels, associated with narrowing of the central spinal canal and narrowing of the passageways where nerves exit on both sides 7. There is disc bulging and ligament thickening at the second, third, and fourth lumbar levels 8. There are old compression fractures in the eighth, tenth, and twelfth thoracic vertebrae	1. A fracture involving the posterior arch of the first cervical vertebra (C1), right side, accompanied by a minor misalignment (subluxation) at the joint between C1 and C2 on the left side (left atlantoaxial joint) 2. A recent compression fracture has been identified in the vertebral body of the third thoracic vertebra (T3) 3. There is compression on the spinal cord at the levels between the third and fourth cervical vertebrae (C3-C4) and between the fourth and fifth cervical vertebrae (C4-C5), attributed to protruding intervertebral discs 4. There is evidence of degenerative spondylolisthesis at the level between the third and fourth cervical vertebrae (C3/C4), a condition where one vertebra slips forward over the one below it due to degeneration 5. Crowned dens syndrome was incidentally detected, characterized by an abnormal enlargement of the soft tissue surrounding the odontoid process and calcific deposits surrounding the odontoid process, likely indicative of calcium pyrophosphate dehydrate (CPPD) crystal deposition 6. Degenerative spondylolisthesis at the level between the fourth and fifth lumbar vertebrae (L4/5) is noted, along with narrowing of the central spinal canal and narrowing of the openings through which spinal nerves exit bilaterally (bilateral neural foraminal stenosis) 7. There is a bulging disc and an enlargement of the ligamentum flavum (a ligament stabilizing the spine) across the levels between the second and fourth lumbar vertebrae (L2-3-4) 8. Previously documented compression fractures have been observed in the vertebral bodies of the eighth, tenth, and twelfth thoracic vertebrae (T8, T10, T12)
Flesch Kincaid	10.4	6.6	9.4	12.3
Gunning Fog	10.4	8.2	13.6	16.5
SMOG index	10.9	8.8	9.5	11.7
ARI	15.0	8.5	9.1	11.3
Average	11.7	8.0	10.4	13.0
Word count	81	141	200	220
Meaning	N/A	2	5	5
Word choice	N/A	3	5	5
Grammar	N/A	5	5	5
Total score	N/A	10	15	15
Meaning	N/A	2	5	5
Word choice	N/A	4	5	5
Grammar	N/A	5	5	5
Total score	N/A	11	15	15

Bold text highlights the transformed parts of the original report

*A case where “spinal cord” was transformed into a more general term “nerves”

**A case where the description of CPPD was transformed and the specific diagnosis disappeared

***A case where the description of a lumbar vertebra was transformed and incorrectly changed to “neck”

### Potential changes in patient management in reports transformed to a 7th-grade reading level

Among the 100 reports transformed to a 7th-grade reading level, 20% (20/100) exhibited potential changes in patient management when compared to the original reports. Notably, all 20 cases occurred within the subset of 32 reports that scored below the median accuracy threshold of 13 points. The inaccuracy patterns identified within these 20 cases were as follows: inappropriate generalization occurred in 40% (8/20), omission in 35% (7/20), and incorrect conversion in 35% (7/20).

## Discussion

### Ethical tensions and risks of oversimplification

There exists reasonable disagreement about the acceptable level of AI-related simplification [39]. Some clinicians and ethicists argue that any simplification introduces risks by potentially omitting crucial details, while others emphasize the ethical obligation to simplify communication to ensure patient autonomy and comprehension. Recognizing this reasonable disagreement underscores the necessity for a cautious, patient-tailored approach.

To assess the ethical dimensions of LLM-based simplification, we focus on three core considerations: (1) the principle of adequate disclosure that underpins informed consent [22, 40], (2) health equity, whether simplified reports disproportionately benefit or disadvantage certain populations [41, 42], and (3) the attribution of responsibility when simplification leads to misinterpretation [43]. These correspond to the core principles of autonomy, justice, and the duty to avoid deception. While these considerations primarily raise ethical questions, they are also frequently reflected in legal frameworks governing healthcare practice. Adequate disclosure is legally mandated to ensure valid informed consent, health equity principles underpin anti-discrimination laws, and responsibility attribution relates directly to professional and institutional liability in cases of miscommunication or harm [25, 44–46].

First, extreme simplification may obscure critical details, undermining a patient’s ability to make an informed choice. On the other hand, excessive complexity can hinder comprehension and exclude patients from meaningful participation in care decisions [23, 25]. Beyond supporting informed decision‑making, providing patients with sufficient and accurate information upholds their fundamental right to know their own health status. This right, recognized in international bioethics instruments such as the WMA Declaration of Lisbon on the Rights of the Patient (Article 7) [47], is ethically independent of decision‑making capacity and remains valid even when clinical decisions are made by others on behalf of the patient. Second, while simplification might help some with lower health literacy, it may exacerbate inaccuracies or misunderstandings for patients from different linguistic or cultural backgrounds [41]. This concern extends to other vulnerable groups. For example, minors may have the maturity to contribute to medical decisions in some cases, yet even when lacking full decision-making authority, most legal systems and international frameworks recognize their right to be informed in a manner appropriate to their level of understanding. Similarly, the UN Convention on the Rights of Persons with Disabilities (2006) [48] affirms that individuals with disabilities have the right to make decisions with assistance and to receive information tailored to their cognitive and linguistic needs. Third, there remains ethical uncertainty around responsibility if an AI-generated simplification misleads a patient. Does liability rest with the developer, the institution, or the physician? Accountability for AI-mediated patient information is increasingly recognized as a shared responsibility. Historically, clinical frameworks have placed ultimate accountability on licensed physicians for any patient-facing communication, even when AI tools are involved [45]. However, recent policies and regulatory frameworks, including those from the American College of Physicians [49] and the European Union Artificial Intelligence Act [46], explicitly extend accountability to AI developers and deploying institutions, recognizing their role in ensuring safe design, validation, and monitoring of high-risk AI systems in healthcare. These sources suggest that responsibility in AI-supported reporting is not solely individual but distributed across multiple actors involved in system design, deployment, and clinical use.

Our analysis of LLM-transformed reports revealed frequent errors at lower reading levels, including overgeneralization (e.g., “spinal cord” misinterpreted as “nerve”, “transient ischemic attack” simplified as “stroke”), omission of critical findings, and incorrect conversions. An example from our dataset illustrates this concern. The original report stated: ‘A new 2.7 cm irregular low-density lesion in segment 3 of liver. Rec) Liver MRI or US of liver to differentiate hepatic metastasis or other focal hepatic lesion.’ The simplified 7th-grade version was: ‘There’s a new bump in part 3 of your liver. The doctor needs more pictures to see what it is.’ The simplification preserved the recommendation for additional imaging but omitted any reference to metastasis and softened the clinical urgency. A patient relying solely on this version might consider follow-up optional, potentially declining essential diagnostic work-up. This subtle shift in tone and specificity illustrates how oversimplification can weaken the legal validity of consent, which requires decisions to be fully informed, explicit, and specific. Similar tone distortions were also observed in other cases, such as hemorrhagic brain metastases being described as benign hemorrhage foci, risking delayed follow-up. These inaccuracies pose not only clinical but also ethical risks—jeopardizing autonomy, compromising informed consent, and introducing medico-legal liability.

### Inherent limitations of current large language models

Our results suggest that, with current LLM capabilities, the 11th-grade reading level constitutes the lowest readability threshold at which clinical accuracy is consistently maintained. This is not an endorsement of 11th-grade readability as ideal or ethically preferred; rather, it reflects the technical limitations of current AI models. The traditionally recommended 7th-grade level remains ethically preferable, especially in terms of health literacy and informed consent, but current models are not yet capable of producing reliably accurate radiology reports at that level.

Notably, accuracy does not improve beyond the 13th-grade level, suggesting that higher complexity offers diminishing clinical value while potentially impeding comprehension. Thus, 11th-grade readability emerges as a current lower bound between accessibility and accuracy, but not as a normative endpoint. Ethical best practices should aim for lower thresholds that preserve accuracy, demanding further model improvement and careful interface design.

Ultimately, we observed that while improving readability can empower patients, oversimplification below the 7th-grade level undermines accuracy. Maintaining high complexity above the 11th-grade level, however, compromises accessibility, especially for patients with limited education or non-native English fluency. Bridging this divide is critical for ethical, equitable communication.

### Ethical and practical strategies for accountable implementation

To mitigate risks in the interim, we recommend a dual-report approach, offering both a simplified summary and a more detailed version. This allows patients to select the level of detail that best suits their needs and ensures transparency about potential omissions. Comprehension testing across formats could inform which content types or patient groups benefit most from simplification. Though some may argue that multiple formats might cause confusion, withholding information based on such assumptions could reflect paternalism. Instead, responsibility lies in offering support, such as follow-up consultations, to ensure patients understand key findings.

Additionally, readability varies by modality. Chest and abdominal reports tended to be simpler, while musculoskeletal and neuroradiology reports were more complex due to specialized terminology. This underscores the importance of context-specific strategies for simplification.

### Transparency and traceability as core ethical safeguards

This dual-report strategy can also mitigate the risk of creating moral crumple zones [50, 51], situations of ambiguous accountability among AI developers, institutions, and healthcare providers, by clearly delineating original and transformed contents. Moreover, overly simplified reports below the 7th-grade level, as observed in this study, might inadvertently deepen epistemic injustice by excluding patients from meaningful participation in interpreting medical knowledge, reinforcing the ethical necessity of balancing readability with accuracy.

Another promising strategy is to enhance explainability by mapping each LLM-generated sentence to its corresponding source in the original radiology report [52–54]. This traceability not only supports transparency and auditability but also reinforces epistemic accountability, reducing the risk of misplaced trust in opaque AI outputs. Recent ethical discourse emphasizes that AI-mediated patient communication should integrate the concept of ‘digital hermeneutics’ [55], where ethical responsibility extends beyond accuracy to encompass interpretative clarity. Digital hermeneutics suggests AI tools must not only simplify medical language but also facilitate patients’ interpretative engagement with medical content. Additionally, emerging ethical frameworks such as ‘AI interpretability ethics’ [56] argue that transparency and comprehensibility of AI processes are ethically mandatory to prevent epistemic marginalization of vulnerable patient populations. Our study aligns with these contemporary ethical perspectives, advocating that radiology report simplification should prioritize interpretative transparency alongside readability, ensuring patients retain epistemic agency in their care decisions. Epistemic traceability is essential to prevent moral crumple zones and clarify distributed responsibility.

### Patient participation and epistemic justice

The increasing use of LLMs like ChatGPT and Gemini in radiology reflects a paradigm shift toward participatory medicine. Traditionally, the technical jargon in radiology reports has excluded patients from meaningful engagement [57]. Patient-centered communication, especially for chronic diseases, improves outcomes through enhanced understanding and shared decision-making [58, 59].

Promoting patient participation through clear and accessible communication not only fulfills ethical obligations of respecting autonomy but also produces tangible medical benefits, including improved adherence to treatment, reduced misunderstandings, and better overall clinical outcomes [60, 61]. Importantly, the lack of direct patient input presents a key ethical gap. Future work should incorporate patient perspectives and comprehension assessments to ensure that simplification efforts align with actual patient needs and expectations. Without such inclusion, there is a risk of epistemic injustice by excluding patients as active participants in the production and interpretation of medical knowledge.

### Study limitations and future research directions

Our study has several limitations. It was conducted in a single tertiary hospital using one LLM (GPT-4 Turbo), which may limit generalizability. Future studies should compare multiple LLMs across diverse populations and clinical contexts. Traditional readability metrics may also overlook semantic nuance, and evaluations by the original radiologists introduce potential bias. Finally, while this study focused on CT and MRI reports, the ethical concerns highlighted here—particularly regarding patient autonomy, informed consent, and clinical accuracy—are likely relevant across radiological practice. However, differences in report length, structure, and clinical communication patterns across other modalities (e.g., ultrasound, mammography, plain radiographs) make the degree of readability shifts and potential LLM-induced errors uncertain. Extrapolation of our findings should therefore be considered hypothetical, warranting future validation in diverse imaging domains.

## Ethical framework and conclusions

To structure our ethical analysis, we applied Beauchamp and Childress’s four-principles framework [25, 62]. Our findings highlight a core ethical tension between respecting autonomy through accessible language and upholding non-maleficence by avoiding inaccuracies. The principle of justice requires vigilance against simplification strategies that disproportionately harm marginalized groups.

In conclusion, the 11th-grade reading level represents the current lower bound for maintaining clinical accuracy in LLM-generated radiology reports, not an ideal, but a pragmatic constraint under present technological conditions. While this level enables some improvement in readability, it remains inaccessible to many patients, reinforcing the need for more sophisticated models and ethical oversight.

Rather than abandoning lower readability targets, healthcare systems should implement layered strategies, such as dual-format reports, explainability mechanisms, and patient engagement efforts, to bridge the gap between technical limitations and ethical aspirations. Ethical communication must be dynamic, inclusive, and grounded in a commitment to both transparency and comprehension, explicitly addressing patients’ relational autonomy.

## Data Availability

The datasets generated and/or analysed during the current study are not publicly available due to institutional data policy but are available from the corresponding author on reasonable request.

## Abbreviations

AI: Artificial intelligence
NLP: Natural language processing
IRB: Institutional review board
LLM: Large language model
CT: Computed tomography
MRI: Magnetic resonance imaging
API: Application programming interface
GPT-4: Generative pre-trained transformer 4
BERT: Bidirectional encoder representations from transformers
PubMed-BERTScore: BERTScore model trained on biomedical texts
SMOG: Simple measure of Gobbledygook
ARI: Automated readability index
ICC: Intraclass correlation coefficient
HIPAA: Health insurance portability and accountability act
